# Role of lipid metabolism in hepatocellular carcinoma

**DOI:** 10.1007/s12672-024-01069-y

**Published:** 2024-06-04

**Authors:** Yulin Cheng, Jun He, Bin Zuo, Yang He

**Affiliations:** 1 MOE Engineering Center of Hematological Disease, Collaborative Innovation Center of Hematology, Jiangsu Institute of Hematology, The First Affiliated Hospital of Soochow University, Cyrus Tang Hematology Center, Soochow University, Suzhou, Jiangsu 215006 China; 2 Department of General Surgery, The First Affiliated Hospital of Soochow University, Suzhou, Jiangsu 215006 China; 3 MOH Key Lab of Thrombosis and Hemostasis, Jiangsu Institute of Hematology, The First Affiliated Hospital of Soochow University, Suzhou, 215006 China

**Keywords:** Hepatocellular carcinoma, Tumor microenvironment, Lipid metabolism, De novo lipogenesis, Fatty acid oxidation, Therapeutic strategy

## Abstract

Hepatocellular carcinoma (HCC), an aggressive malignancy with a dismal prognosis, poses a significant public health challenge. Recent research has highlighted the crucial role of lipid metabolism in HCC development, with enhanced lipid synthesis and uptake contributing to the rapid proliferation and tumorigenesis of cancer cells. Lipids, primarily synthesized and utilized in the liver, play a critical role in the pathological progression of various cancers, particularly HCC. Cancer cells undergo metabolic reprogramming, an essential adaptation to the tumor microenvironment (TME), with fatty acid metabolism emerging as a key player in this process. This review delves into intricate interplay between HCC and lipid metabolism, focusing on four key areas: de novo lipogenesis, fatty acid oxidation, dysregulated lipid metabolism of immune cells in the TME, and therapeutic strategies targeting fatty acid metabolism for HCC treatment.

## Background

Hepatocellular carcinoma (HCC), a prevalent malignancy, significantly contributes to cancer-related mortality worldwide [1]. It ranks sixth in incidence and fourth in cancer-related deaths, with a relative 5-year survival rate of around 18% [1, 2]. The prevalence of HCC is increasing, and between 2020 and 2040, the number of new cases is expected to rise by 55.0%, with approximately 1.4 million new diagnoses [3]. The diagnosis of HCC peaks in populations between the ages of 60 and 70, with a two- to four-fold higher incidence rate in men than in women [1]. Virus infection, including hepatitis B and C, and alcohol consumption present an extensive mortality and risk for HCC [4]. Recently, fatty liver-related diseases, including nonalcoholic fatty liver disease (NAFLD) and nonalcoholic steatohepatitis (NASH), have been prove to be an increasingly dominant underlying aetiology of HCC [5, 6].

While carbohydrate metabolism dysregulation has historically dominated the understanding of cancer's metabolic reprogramming (a hallmark of the disease) [7], the significance of altered lipid metabolism in cancer is gaining increasing recognition [8, 9]. Cancer cells require substantial energy during development to adapt the demands of survival, growth, proliferation, invasion, metastasis, and it is in support of the reprogrammed lipid metabolism [10]. These adaptations specifically refer to enhanced lipid catabolism and anabolism for energy and harness lipid metabolism to regulate the stromal and immune cells to resist therapy and promote relapse [11]. In recent years, alterations in lipid metabolism in cancer cells have received increasingly attention and research on breast cancer [12] and colorectal cancer [13].

**Fig. 1:**
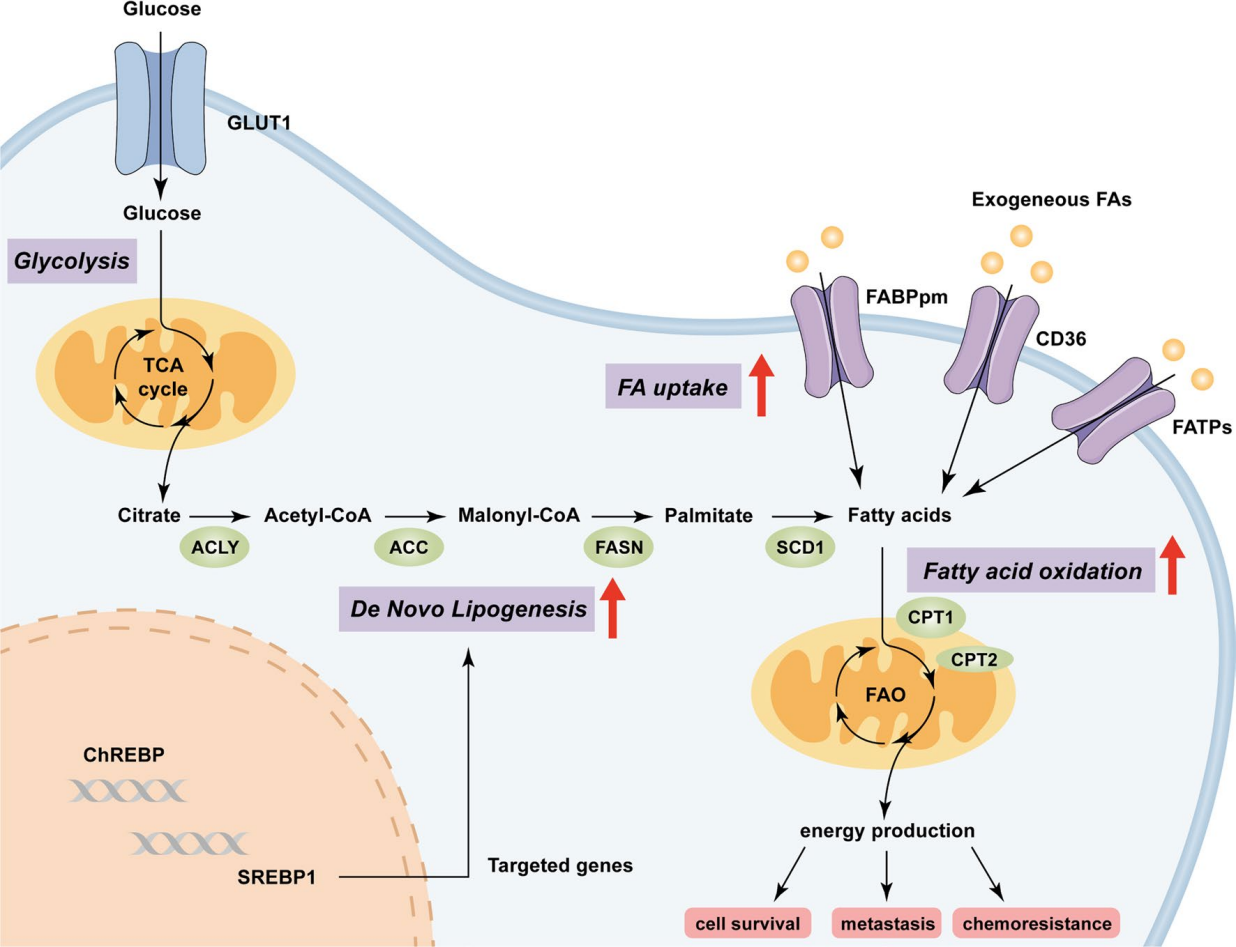
Lipid metabolism in hepatocellular carcinoma. Exogenous FAs are transported into the cytoplasm via specialized transporters such as CD36, FATPs, and FABPpm. In cancer cells, FAs are also synthesized through enhanced de novo lipogenesis (DNL), which promotes the expression of DNL core enzymes by SREBP1. FAs and their synthetic products subsequently enter the mitochondria to produce NADPH and acetyl-CoA through β-oxidation for energy production to promote cell survival, metastasis, and chemoresistance. FAs, fatty acids; GLUT1, glucose transporter 1; TCA, tricarboxylic acid; ACLY, ATP-citrate lyase; FABPpm, plasma membrane fatty acid-binding proteins; FATPs, fatty acid transport protein family; ACC, acetyl-CoA carboxylase; FASN, fatty acid synthase; SCD1, stearoyl-CoA desaturase1; CPT1, carnitine palmitoyl transferase 1; CPT2, carnitine palmitoyl transferase 2; FAO, fatty acid oxidation; SREBP, cleavage activating protein; ChREBP, carbohydrate response element binding protein

Accumulated studies focused primarily on the liver because of its importance in whole-body cholesterol and fatty acids (FAs) homeostasis [14]. Studies of metabolomics and lipidomics showed that fatty acids significantly changed in abundance in tumorigenesis, and an agreement between dynamic changes observed in circulating fatty acids with the liver deterioration and progression towards HCC, which is also associated with tumor size [15, 16]. The dysregulation of fatty acid metabolism aberrantly activated signaling pathways by altering the lipid-metabolizing enzyme expression in HCC cells, thereby favoring cell survival, metastasis, chemoresistance in HCC (Fig. 1). While existing literature have offered valuable insights into metabolic alterations in HCC [17–20], this review aims to further enrich the existing knowledge by focusing on the most recent advancements on the evolving landscape of lipid metabolism in HCC, including de novo lipogenesis (DNL), fatty acid oxidation (FAO), aberrant lipid metabolism in immunocytes in the HCC microenvironment and therapeutic strategies targeting lipid metabolism for HCC treatment.

## Lipid provision in HCC

FAs are essential for cell survival, serving not only as fundamental structural components of cell membranes but also as vital energy donors. Most cells obtain FAs either from exogenous dietary sources or from de novo lipogenesis or de novo fatty acid synthesis by which fatty acids are synthesized from excess carbohydrates. These FAs can then be incorporated into triglycerides (TGs) for energy storage [21]. While dietary sources as well as lipid synthesis by liver are major ways to obtain lipids, several cancers have been reported to reactivate DNL, making them more independent of exogenous lipids [22].

### De novo lipogenesis

DNL, the biosynthesis of FAs from non-lipid precursors, exhibits high activity in metabolically robust tissues such as the liver, adipose tissue, and skeletal muscle [21, 23, 24]. Notably, cancer cells exhibit a unique characteristic: they aberrantly activate the DNL pathway even with exogenous lipid sources [25, 26]. Multiple studies have demonstrated dysregulated DNL in HCC. Most recently, a comprehensive analysis combining metabolomics, lipidomics and gene expression data revealed significantly elevated DNL in HCC compared to healthy liver tissue [27]. Growth differentiation factor 11 (GDF11) was shown to increase lipid accumulation and DNL in cancer cells, but not in healthy hepatocytes [28]. Additionally, LINC00958, a lipogenesis-related long non-coding RNA, upregulates hepatoma-derived growth factor in HCC, ultimately promoting lipogenesis [29]. Acyl-CoA thioesterase 9 also contributes to DNL upregulation in HCC [30].

Furthermore, core enzymes of DNL, including ATP-citrate lyase (ACLY), acetyl-CoA carboxylase (ACC), fatty acid synthase (FASN) and Stearoyl-CoA desaturase (SCD), are altered in HCC [31]. For instance, FASN has been correlated with tumor initiation. Loss of FASN is sufficient to delay tumor initiation, and this is largely correlated with increased sterol regulatory element-binding proteins (SREBPs) activity, which results in the upregulation of genes involved in DNL and cholesterol biosynthesis [32]. In HCC cells, activation of mitochondrial fission has been shown to upregulate the acetylation level of SREBP1 by suppressing nicotinamide adenine dinucleotide (NAD +)/Sirtuin 1 (SIRT1) signaling. Eventually, elevated SREBP1 upregulates the expression of FASN, ACC and elongation of very long chain fatty acid protein 6 (ELOVL6), all of which are involved in DNL [33]. In the progression of HCC, USP7/ZNF638 axis, correlating with ubiquitination, selectively increases the cleavage of SREBP1c, and subsequently regulates lipogenesis-associated enzymes, including ACC, FASN, and SCD [34]. It is also notable that ACC is bound to upstream stimulatory factor 1 (USF1)-induced fatty acid synthesis-related lncRNA (FASRL), leading to enhanced fatty acid synthesis and lipid accumulation to subsequently aggravate HCC [35]. Moreover, inhibition of ubiquitin-specific protease 22 (USP22) reduces de novo fatty acid synthesis and mitigate HCC tumorigenesis, which is largely achieved through suppressing ACC and ACLY through inhibiting the expression of peroxisome proliferators-activated receptor (PPAR) [36]. ACLY, a potent regulator of Wnt/β-catenin signaling, is also essential for modulating liver tumor-initiating cells stemness and metastasis in HCC [37]. Lipidomic analysis has revealed that alterations in the lipid composition of HCC cells in response to matrix stiffness are mediated by SCD1. The expression of SCD1 affected the MUFA/SFA ratio, which could be attributed to the impact on membrane fluidity, ultimately promoting HCC invasion and metastasis [38].

### Transcriptional regulation of DNL

**Fig. 2:**
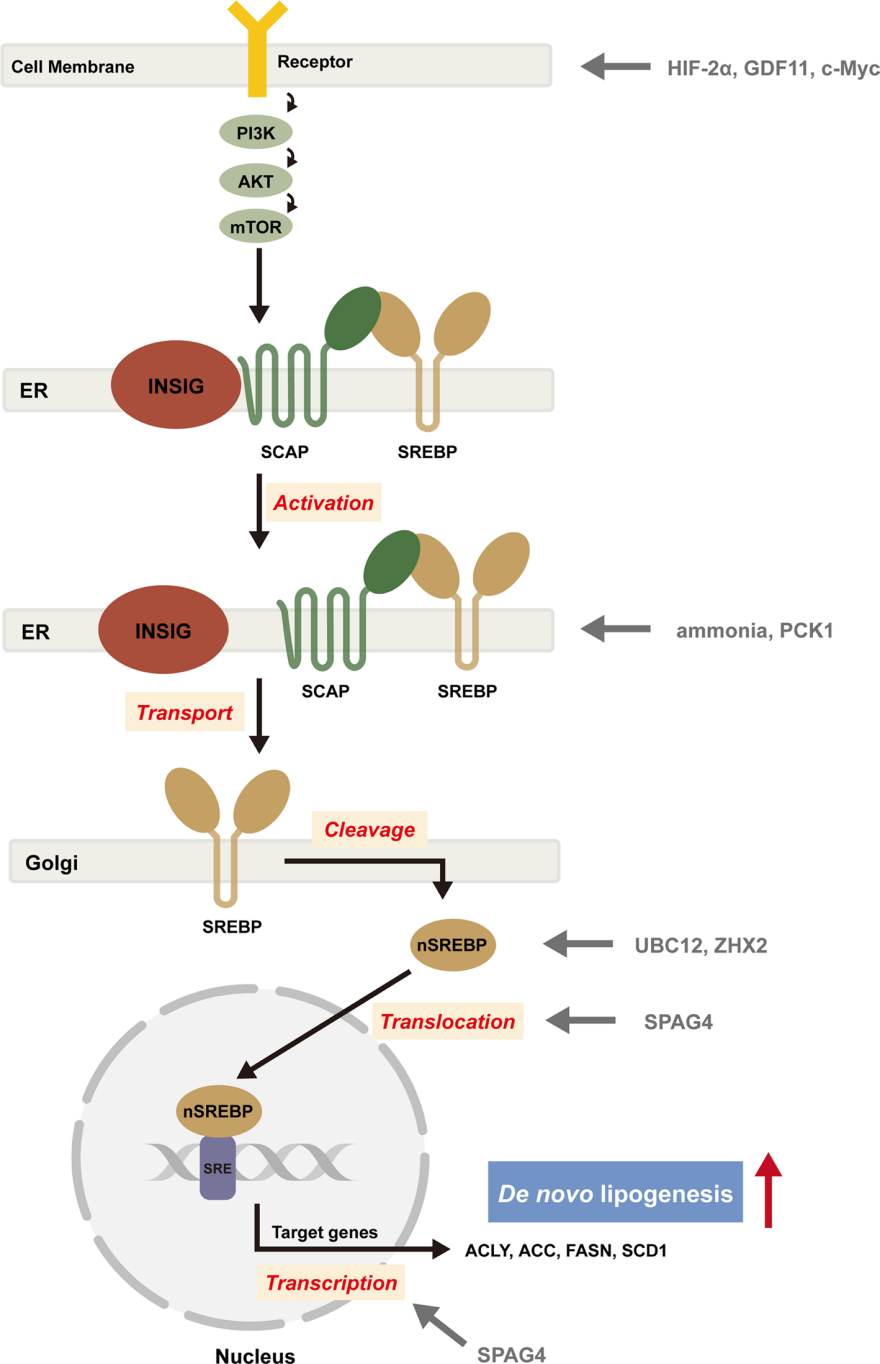
Transcriptional regulation of de novo lipogenesis in HCC. SREBPs serve as key regulators of lipogenesis at the transcriptional level by facilitating the expression of genes involved in de novo lipogenesis, such as ACLY, ACC1, FASN and SCD1. Evidence also suggests that the activity of SREBPs is modulated by the phosphatidylinositol 3-kinase (PI3K)/AKT/mTOR pathway. SREBPs, sterol regulatory element-binding proteins; ACC1, acetyl-CoA carboxylases 1; ACLY, ATP-citrate lyase; SCD1, stearoyl-CoA desaturase-1; FASN, fatty acid synthase; SCAP, SREBP cleavage activating protein; ER, endoplasmic reticulum; INSIG, insulin induced gene; Golgi, Golgi complex; SPAG4, sperm associated antigen 4; UBC12, ubiquitin conjugating enzyme E2 M; ZHX2, zinc fingers and homeoboxes 2; PI3K, Phosphatidylinositol 3-kinase; mTOR, mammalian target of rapamycin

DNL is primarily regulated by the activation of SREBPs at the transcriptional level (Fig. 2). SREBPs serve as pivotal factors in initiating lipogenesis and are crucial for maintaining intracellular lipid homeostasis [39]. Multiple studies have been shown that DNL can be suppressed by targeting the activation of SREBP1 [40, 41]. The SREBP family comprises three distinct members: SREBP1a, SREBP1c, and SREBP2, each with different roles in driving lipid synthesis and uptake [42]. The significant involvement of SREBP1 in regulating cancer metabolism has been widely recognized, extending to various cancer types such as squamous cell carcinomas, colorectal cancer, and pancreatic cancer [43–45]. In HCC, the activation of SREBP1 has also been observed, which is largely responsible for tumor proliferation rate and poor prognosis [46]. Moreover, ammonia generated from glutamine promotes lipogenesis through regulating SREBPs. Specifically, ammonia activates the dissociation of N-glycosylated SREBP-cleavage-activating protein (SCAP) from insulin-induced gene (INSIG) in the endoplasmic reticulum, leading to SREBPs translocation and subsequent lipogenic gene expression [47]. Furthermore, phosphoenolpyruvate carboxykinase 1 (PCK1), the rate-limiting enzyme in gluconeogenesis, also facilitates lipogenesis by promoting the translocation of the SCAP-SREBP complex to the Golgi apparatus, activating SREBPs and ultimately promoting tumor cell proliferation and tumorigenesis [48]. Neddylation of SREBP1 by UBC12 prolongs SREBP1 stability and decreases ubiquitination, which drives HCC proliferation, migration and invasion in vitro and in vivo [49]. In addition, long chain acyl CoA synthetase 4 (ACSL4) plays a crucial role in modulating DNL by promoting the accumulation of intracellular triglycerides, cholesterols, and lipid droplets in HCC. This effect is primarily mediated through upregulation of SREBP1 and its downstream lipogenic enzymes via c-Myc, all contributing to HCC cell proliferation and metastasis [50]. Zinc fingers and homeoboxes 2 (ZHX2) inhibits DNL in HCC, whereby ZHX2 activates miR-24-3p transcriptionally to target SREBP1c and leads to its degradation and consequent HCC progression [51]. Sperm associated antigen 4 (SPAG4) is up-regulated in HCC tissues. It enhances SREBP1 expression, nuclear translocation, and transcriptional activity by interacting with lamin A/C through its N-terminal region [52].

Phosphatidylinositol 3-kinase (PI3K)/AKT pathway, a well-established regulator of cell survival and proliferation, has also been implicated in the activation of SREBPs [53]. This pathway promotes cell growth through the activation of its downstream target, the target-of-rapamycin complex 1 (TORC1). Conversely, inhibition of mTORC1 using rapamycin, for example, prevents the nuclear translocation of SREBP1 and the subsequent expression of its downstream genes [54]. The PI3K/AKT/mTORC1 pathway has been shown to suppresses ferroptosis in cancer cells via downstream SREBP1/SCD1-mediated lipogenesis [55]. In a hypoxic microenvironment, hypoxia-inducible transcription factor-2α (HIF-2α) is upregulated and then activates the PI3K/AKT/mTOR pathway. This cascade ultimately promotes lipid synthesis, exacerbating lipid accumulation, thereby fostering growth, migration, invasion and angiogenesis in NAFLD and HCC [56]. Moreover, GDF11 significantly reduces cholesterol and triglycerides in HCC cells and inhibits mTOR signaling pathway, resulting in a defective metabolism of cancer cells [57]. Furthermore, it also suggests that c-Myc-dependent hepatocarcinogenesis is associated with FASN, a pivotal downstream effector of mTORC1 [58].

### Exogenous uptake of lipids

Despite favoring lipogenesis for endogenous fatty acid synthesis, cancer cells retain a significant dependence on exogenous FAs for their progression. External FAs enter cells through various lipid transporters including CD36, fatty acid transport protein family (FATPs), and plasma membrane fatty acid-binding proteins (FABPpm) [59]. HCC cells undergo significant metabolic reprogramming to utilize and satisfy their high energy demands [60]. Upregulation of CD36 accelerates HCC progression by promoting FA absorption and is associated with increased invasiveness, such as epithelial-to-mesenchymal transition (EMT) [61, 62]. Notably, CD36-mediated uptake of oxidized LDL (ox-LDL) triggers a pathway involving Nogo-B and CEBPβ expression, ultimately reprogramming ox-LDL metabolism and driving cancer development in NAFLD-associated HCC [63]. Conversely, miR-3180 acts as a tumor suppressor by targeting CD36, thereby inhibiting both DNL and exogenous FA uptake and hindering HCC growth and metastasis [64]. It is reported that CD36 is upregulated in cancer-associated fibroblasts (CAFs) in comparison to peri-tumor fibroblasts (PTFs) in HCC, resulting in the altered lipid metabolism in tumor cells [65]. Interestingly, FATP4 shows the opposite behavior, being overexpressed in HCC and associated with poor prognosis. This overexpression facilitates FA uptake and protects against both lipid peroxidation and ferroptosis [66].

## Fatty acid oxidation in HCC

Initiation of FAO necessitates the prior activation of FAs to fatty acyl-CoA esters by a family of acyl-CoA synthetases. Carnitine palmitoyl transferase 1 (CPT1) then catalyzes the conversion of these fatty acyl-CoA esters to acyl-carnitine derivatives, facilitating their import into the mitochondrial matrix via carnitine-acylcarnitine translocase. Within the matrix, carnitine is liberated by CPT2, regenerating fatty acyl-CoA substrates. Through iterative cleavage, these substrates are converted to acetyl-CoA, which fuels the Krebs cycle and generates reducing equivalents for ATP synthesis [67]. Increasing evidence has shown that abnormal FAO is involved in various aspects of oncogenesis, including proliferation, survival, stemness, drug resistance or metastasis [68, 69]. Dissecting the multi-faceted roles of FAO would be instrumental in our understanding of HCC.

### Fatty acid transport

Carnitine plays a crucial role in transporting long-chain fatty acids (LCFAs) into the mitochondria for β-oxidation, the process generating energy for cellular functions [70]. This intricate process involves two key enzymes: CPT1 on the outer mitochondrial membrane, which attaches carnitine to LCFAs, and CPT2 on the inner membrane, responsible for cleaving the carnitine molecule and releasing the fatty acid [71]. CPT is involved in various cellular processes, including immune escape and metastasis, by regulating FAO in cancer cells [72, 73].

Interestingly, recent studies have linked altered carnitine metabolism to tumor progression in HCC. One study revealed that suppressed mitochondrial fission has been shown to enhance FAO through upregulating CPT1A, significantly promoting the proliferation and metastasis, which is largely attributed to the nicotinamide adenine dinucleotide (NAD +)/Sirtuin 1 (SIRT1) signaling [33]. The miR-377-3p/CPT1C axis is necessary for HCC, as miR-377-3p suppresses FAO by preventing the transport of FAs and reduces further energy production in cancer cells through repression of CPT1C [74]. It is also notable that CPT1A which regulates lipid accumulation, is essential for lipid homeostasis in HCC. Overexpressed CPT1A in cancer cells contribute to avoid lipotoxicity in lipid-rich environment [75]. Besides, CPT2 suppressed by the cell-cycle regulator E2F1 and E2F2 contributes to generating the lipid-rich environment [76]. In addition, the solute carrier family 27 member 5 gene (SLC27A5) is associated with FAs uptake into cells and bile acid metabolism, while exerting a tumor-suppressive effect by suppressing TXNRD1 expression via the KEAP1/NRF2 pathway in HCC [77]. Moreover, solute carrier family 25 member 20 (SLC25A20) inhibits HCC growth and metastasis by suppression of G1-S cell transition, EMT, and induction of cell apoptosis. And SLC25A20, which is downregulated in HCC, promotes HCC growth and metastasis by suppressing FAO [78].

### Fatty acid catabolism

Within the mitochondria, fatty acyl-CoA undergoes sequential cleavage via a four-step enzymatic cycle to generate acetyl-CoA. This repetitive process is mediated by acyl-CoA dehydrogenase, hydroxyacyl-CoA dehydrogenase, enoyl-CoA hydratase and 3-ketoacyl-CoA thiolase (3-KAT). Finally, acetyl-CoA fuels the tricarboxylic acid (TCA) cycle for cellular energy production [79]. Studies have shown that elevated caveolin-1 (CAV1) expression in HCC inhibits FAO [80]. Mechanistically, CAV1 upregulation promotes the nuclear accumulation of SREBP1. SREBP1, in turn, suppresses medium-chain acyl-CoA dehydrogenase (ACADM), a key enzyme in β-oxidation. This sequential repression ultimately leads to increased HCC cell aggressiveness [80]. These findings highlight the potential importance of the CAV1/SREBP1/ACADM axis in HCC progression. Long-chain acyl-CoA dehydrogenase (ACADL) catalyzes the initial step of β-oxidation for long-chain fatty acyl-CoA substrates within the mitochondria. Interestingly, research suggests that ACADL functions as a tumor suppressor in HCC by inhibiting metastasis via the signal transducer and activator of transcription 3 (STAT3)/Matrix Metalloproteinase 14 (MMP14) pathway [81].

## Aberrant lipid metabolism of immunocytes in HCC

**Fig. 3:**
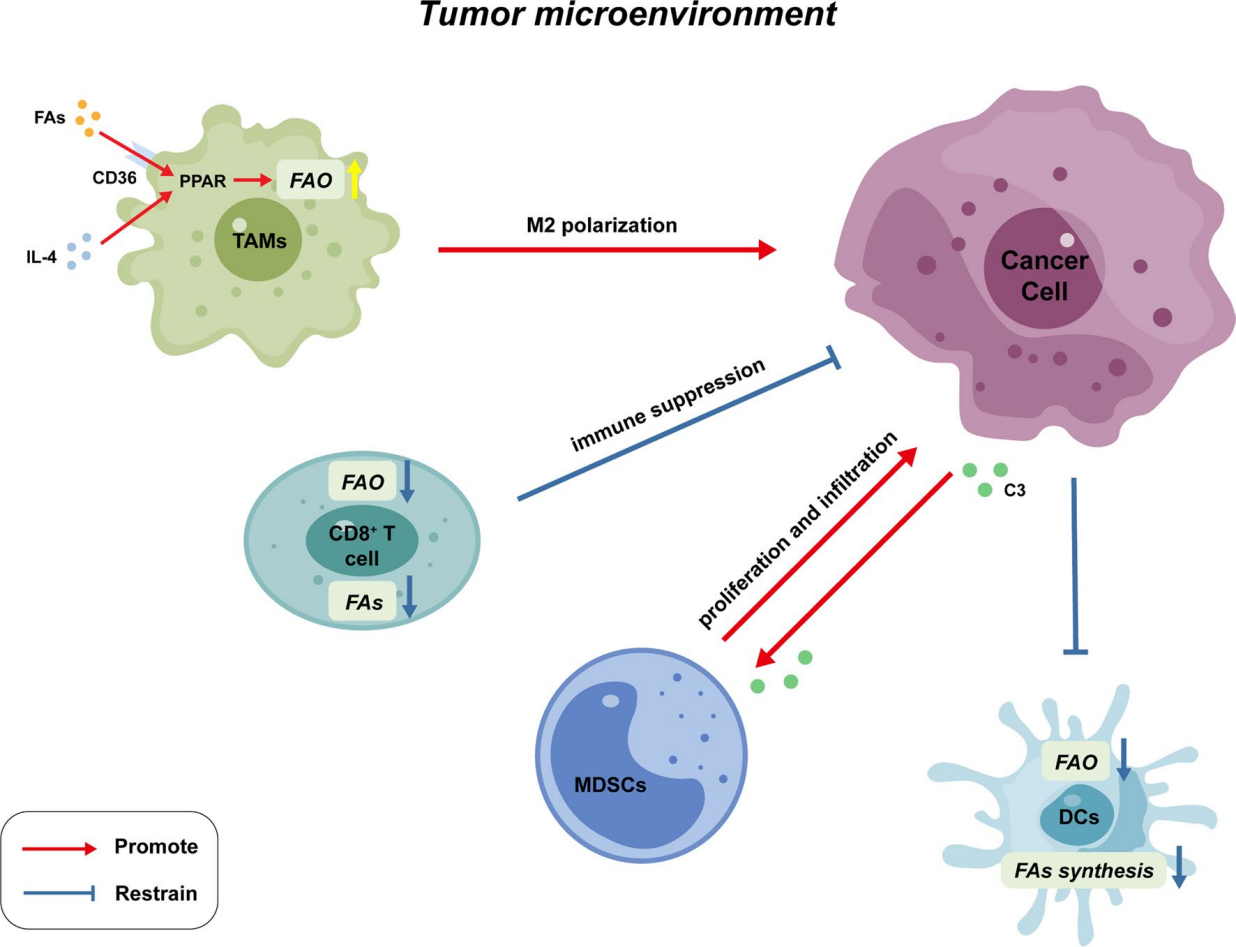
Altered lipid metabolism in the tumor microenvironment affects anti‐/pro‐tumor functions of immune cells. In the TME of HCC, tumor cells can alter the metabolism of the tumor-associated immune cells, which, in turn, interferes with tumor cells. TME, tumor microenvironment; TAMs, tumor-associated macrophages; FAO, fatty acid oxidation; MDSCs, myeloid-derived suppressor cells; DCs, dendritic cells; FAs, fatty acids

The liver serves as a critical frontline immune organ, where intricate interactions between its diverse immune cell populations orchestrate a delicate balance between immunity, tolerance, and overall tissue health [82]. Similarly, intercellular metabolic communication within the tumor microenvironment (TME) is emerging as a significant contributor to cancer development, particularly in the context of immune cell response [83]. This complex interplay between immune cells and tumor cells plays a crucial role in HCC progression (Fig. 3).

### Tumor-associated macrophages (TAMs)

Lipid metabolism also play a crucial role in generating TAMs in the TME [84]. Macrophages are the innate immunocytes involved in various biological processes, including host defense, homeostasis and disease progression. The surrounding microenvironment induces the polarization of M1 and M2 macrophage phenotypes [85]. TAMs show similar functions as M2s, as some researchers demonstrate TAMs as a polarized M2 macrophage population [86]. In the TME, TAMs survive nutrient- and oxygen-limited environment and acquire tumor-promoting capabilities through metabolic reprogramming [87]. High M2 TAMs count is often associate with an inferior outcome [88]. Fatty acid synthesis and oxidation in TAMs are activated and contribute to tumor growth.

TAMs are characterized by high expression of the scavenger receptor CD36, facilitating lipid accumulation and subsequent utilization through FAO for energy production. This metabolic signature is critical for TAM generation, function, and polarization, particularly towards the M2 phenotype, ultimately promoting tumorigenesis [89]. Notably, receptor-interacting protein kinase 3 (RIPK3) has emerged as a key regulator of this process, promoting both M2 TAM accumulation and polarization within the tumor microenvironment. RIPK3 deficiency in TAMs resulted in a significant suppression of caspase-1-mediated PPAR cleavage. This, in turn, promoted M2 polarization within TME in conjunction with elevated fatty acid metabolism, encompassing FAO, suggesting that the accumulation and altered lipid metabolism of M2 TAMs in the TME accelerates HCC growth [90]. Furthermore, S100A4, an M2 activator of macrophage polarization induced by IL-4, controls the upregulation of PPAR-γ, which is required to facilitate FAO and enhance FA absorption by upregulating CD36. Collectively, macrophagic S100A4 enhances the polarization of protumor macrophages as a determinant of PPAR-γ-dependent FAO induction [91]. In addition, prostaglandin E2 (PGE2) was found to promote the dissociation of KLF transcription factor 6 (KLF6) transcription factor from the miR-520d promoter, leading to decreased miR-520d expression and subsequent upregulation of ubiquitin-like with PHD and ring finger domains 1 (UHRF1). This cascade ultimately culminates in HCC progression. These findings establish a reciprocal positive feedback loop between TAMs and HCC cells, fostering a self-reinforcing tumorigenic microenvironment within the tumor [92].

### T cells

T cells are recognized as crucial components of the immune system, particularly in the context of anti-tumor immunity. Recent advancements in immune cell-based analyses have yielded three distinct T cell clusters characterized by unique features. These findings promote the development of personalized therapeutic strategies aimed at improving clinical outcomes and optimizing patient management [93]. Studies has revealed that T cells exhibit different metabolic requirements in the TME [94]. Acyl-CoA:cholesterol acyltransferase (ACAT) inhibition alters the lipid composition of CD8^+^ T cells, thereby augmenting both TCR signaling and TCR-independent bioenergetic pathways. Consequently, this approach enhances the efficacy of PD-1 blockade therapy and bolsters the functional activity of TCR-gene-modified T cells. Given the critical role of T cells in restraining tumors and viral infections, targeting ACAT represents a promising therapeutic strategy for HBV-associated HCC [94]. Furthermore, the natural killer T (NKT) cell is characterized with both adaptive and innate immune cells. The altered tumor lipid metabolic profile and accumulated lipids and fatty acids that favor evading anti-tumor immunity may impair the immune-modulatory function lipid antigens recognized by CD1d-restricted T cells [95]. In addition, NKT dysfunction has been found to be mediated by mTORC1/SREBP2/cholesterol in the tumor-promoting NAFLD liver microenvironment. Specifically, obesity-induced cholesterol accumulation selectively activated by mTORC1/SREBP2 signaling also impairs NKT cell-mediated antitumor surveillance in the liver [96]. Aberrant lipid metabolism, accumulated long-chain acylcarnitine, especially palmitoyl-carnitine and stearoyl-carnitine, inhibit iNKT cell expansion and promote senescence [97]. As mentioned above, further studies should shed light on the role of T cell with abnormal lipid metabolism in HCC.

### Other immune cells

Beyond the aforementioned immunocytes, dysregulated lipid metabolism extends to myeloid-derived suppressor cells (MDSCs) and dendritic cells (DCs), potentially contributing to tumor progression. MDSC accumulation has been linked to HCC metastasis, possibly through upregulation of PD-L1 and chemokine CCL2 [98]. Piwi like RNA-mediated gene silencing 1 (PIWIL1), overexpressed in HCC, appears to initiate intercellular metabolic crosstalk, significantly promoting HCC proliferation and growth through the utilization of FAs as a primary energy source [99]. Furthermore, observations suggest that PIWIL1-induced FAO may promote MDSC accumulation within liver tissue and PIWIL1-mediated immunosuppression. This immunosuppression may be dependent on PIWIL1-induced secretion of complement C3, which activates p38 MAPK signaling in MDSCs, ultimately initiating the expression of the immunosuppressive cytokine IL-10 [99]. A reduction in fatty acid synthesis and mitochondrial metabolism has been observed in DCs as a result of exposure to α-fetoprotein (AFP), a protein secreted by HCC cells. And AFP have been shown to downregulate SREBP1 and PGC1-α, which are involved in metabolic regulation. Importantly, PGC1-α leads to impaired ability to stimulate antigen-specific effector functions [100].

## Targeting lipid metabolism for HCC treatment

The hypoxic and nutrient-deprived tumor microenvironment in HCC compels cancer cells to rely heavily on lipid metabolism for survival, making it a promising therapeutic target. The potential of targeting lipid metabolism for HCC diagnosis, treatment has been comprehensively reviewed in previous works [17–20], the present review complements existing literature by specifically focusing on recent advancements in potential lipid metabolism-targeted therapies for HCC treatment within the past three years (Table 1).

**Table 1:** Lipid metabolism-targeted strategies for HCC therapy

Targets	Agents	Mechanisms	References
SREBP	Dipyridamole	Inhibition of SREBP processing	[101]
	Cinobufotalin (CBF)	Inhibition of the expression of SREBP	[102]
	Bufalin	Inhibition of the expression of SREBP1	[103]
	Metformin	Inhibition of the activities of SREBPs	[104]
ACADL	Verteporfin	YAP inhibitor; Inhibition of FAO	[105]
SOAT1/CPT1A	Avasimibe and etomoxir	Inhibition of FAO	[75]
PPAR	Oroxyloside (OAG)	Dual agonist of PPARγ/ɑ; Induction of Glycolipid Metabolism Switch	[106]
FASN	Orlistat	Inhibition of FA synthesis	[52]
	TVB3664	Inhibition of FA synthesis	[107]
	Curcumin	Inhibition of FA synthesis	[108]
	Osthole	Inhibition of FA synthesis	[109]

SREBPs, sterol regulatory element-binding proteins; ACADL, long-chain acyl-CoA dehydrogenase; FASN, fatty acid synthase; SOAT, sterol o-acyltransferase; CPT, carnitine palmitoyl transferase; FAO, fatty acid oxidation; PPAR, peroxisome proliferators-activated receptor; FASN, fatty acid synthase

### Targeting SREBP1 and lipogenesis

SREBP1, a master regulator of lipogenesis, has been extensively investigated as a potential therapeutic target in HCC. Cinobufotalin (CBF), extracted from the skin secretion of the giant toad, has been shown to selectively inhibit SREBP1 expression and its interaction with sterol regulatory elements (SREs), leading to suppression of lipogenic enzyme expression and HCC tumorigenesis [102]. Similarly, bufalin exerts anti-tumor effects by downregulating the expression of SREBP1 and lipogenesis-related genes [103]. Dipyridamole, a clinically used phosphodiesterase inhibitor, potentiates statin-induced tumor growth inhibition by directly targeting SCAP and INSIG, thereby inhibiting SREBP processing [101].

### Targeting fatty acid oxidation (FAO)

Verteporfin, a YAP inhibitor, effectively suppresses the growth of HCC organoids with low ACADL expression, highlighting the potential of targeting FAO for HCC therapy [105]. Furthermore, the combination of sterol o-acyltransferase1 (SOAT1) and CPT1A inhibitors, avasimibe and etomoxir, exhibits synergistic anticancer effects in HCC models [75].

### Metabolic reprogramming and novel therapeutic strategies

Oroxyloside (OAG), a dual PPAR-γ/α agonist, represents a novel therapeutic approach for HCC. OAG induces a metabolic switch by regulating glycolipid metabolic enzymes, both PPAR-dependent and independent, leading to increased ROS levels, cell cycle arrest, and ultimately, reduced cell proliferation [106]. Metformin, widely used for diabetes, has also demonstrated beneficial effects in HCC by inhibiting SREBP activity through targeting SLC25A47 and regulating lipid homeostasis [104].

### Targeting fatty acid synthase (FASN)

Orlistat, a lipid synthesis inhibitor, showed antitumor effects in HCC, reversing SPAG4-mediated oncogenic effects [52]. TVB3664, a FASN inhibitor, shows promising therapeutic efficacy in combination with sorafenib and cabozantinib in preclinical HCC models, addressing the limitations of current treatment options [107]. Additionally, natural compounds like curcumin and osthole exhibit anti-HCC effects by targeting FASN [108, 109].

## Conclusions and future perspectives

Recent advancements in understanding the rewiring of lipid metabolism in cancer cells have significantly broadened our knowledge of its role in HCC progression. While dysregulated lipid metabolism is a hallmark of HCC, translating promising preclinical findings into effective clinical therapies remains challenging. Careful consideration is crucial regarding patient heterogeneity, as HCC patients exhibit diverse lipid profiles, necessitating personalized approaches. Additionally, modulating lipid metabolism may have unintended effects on other organs or metabolic pathways. For example, FASN inhibitors are known to induce rapid weight loss due to their impact on fatty acid oxidation [110]. Furthermore, solely targeting fatty acid metabolism might be insufficient due to the metabolic adaptability of tumors. Through metabolic reprogramming, tumors can activate alternative pathways to ensure survival. It is crucial to consider the intricate regulatory networks governing lipid metabolism in HCC and its interplay with other cell types within the TME. For instances, lipid metabolism-targeted drugs may exhibit conflicting effects on T cells [111]. Therefore, exploring more specific lipid-targeting agents or combining them with existing therapies like chemotherapy and immunotherapy could potentially enhance treatment efficacy. Notably, most agents lack clinical evaluation, necessitating further research to develop more human-relevant models and establishing stronger correlations between preclinical and clinical outcomes by exploring personalized medicine strategies, developing better biomarkers for patient selection, and conducting well-designed clinical trials.

## Data Availability

No new data were created or analysed in this study.

## References

[1] Vogel A, Meyer T, Sapisochin G (2022). Hepatocellular carcinoma. Lancet.

[2] Brown ZJ, Tsilimigras DI, Ruff SM (2023). Management of hepatocellular carcinoma: a review. JAMA Surg.

[3] Rumgay H, Arnold M, Ferlay J (2022). Global burden of primary liver cancer in 2020 and predictions to 2040. J Hepatol.

[4] Akinyemiju T, Abera S, Ahmed M (2017). The burden of primary liver cancer and underlying etiologies from 1990 to 2015 at the global, regional, and national level: results from the global burden of disease study 2015. JAMA Oncol.

[5] Huang DQ, El-Serag HB, Loomba R (2021). Global epidemiology of NAFLD-related HCC: trends, predictions, risk factors and prevention. Nat Rev Gastroenterol Hepatol.

[6] Ioannou GN (2021). Epidemiology and risk-stratification of NAFLD-associated HCC. J Hepatol.

[7] Boroughs LK, Deberardinis RJ (2015). Metabolic pathways promoting cancer cell survival and growth. Nat Cell Biol.

[8] Gao L, Xu Z, Huang Z (2020). CPI-613 rewires lipid metabolism to enhance pancreatic cancer apoptosis via the AMPK-ACC signalling. J Exp Clin Cancer Res.

[9] Liu F, Ma M, Gao A (2021). PKM2-TMEM33 axis regulates lipid homeostasis in cancer cells by controlling SCAP stability. Embo j.

[10] Bian X, Liu R, Meng Y (2021). Lipid metabolism and cancer. J Exp Med.

[11] Martin-Perez M, Urdiroz-Urricelqui U, Bigas C (2022). The role of lipids in cancer progression and metastasis. Cell Metab.

[12] Dai W, Xiang W, Han L (2022). PTPRO represses colorectal cancer tumorigenesis and progression by reprogramming fatty acid metabolism. Cancer Commun (Lond).

[13] Liu S, Sun Y, Hou Y (2021). A novel lncRNA ROPM-mediated lipid metabolism governs breast cancer stem cell properties. J Hematol Oncol.

[14] Alves-Bezerra M, Cohen DE (2017). Triglyceride Metabolism in the Liver. Compr Physiol.

[15] Muir K, Hazim A, He Y (2013). Proteomic and lipidomic signatures of lipid metabolism in NASH-associated hepatocellular carcinoma. Cancer Res.

[16] Ismail IT, Elfert A, Helal M (2020). Remodeling lipids in the transition from chronic liver disease to hepatocellular carcinoma. Cancers (Basel).

[17] Paul B, Lewinska M, Andersen JB (2022). Lipid alterations in chronic liver disease and liver cancer. JHEP Rep.

[18] Du D, Liu C, Qin M (2022). Metabolic dysregulation and emerging therapeutical targets for hepatocellular carcinoma. Acta Pharm Sin B.

[19] Alannan M, Fayyad-Kazan H, Trézéguet V (2020). Targeting lipid metabolism in liver cancer. Biochemistry.

[20] Pope ED, Kimbrough EO, Vemireddy LP (2019). Aberrant lipid metabolism as a therapeutic target in liver cancer. Expert Opin Ther Targets.

[21] Ameer F, Scandiuzzi L, Hasnain S (2014). De novo lipogenesis in health and disease. Metabolism.

[22] Snaebjornsson MT, Janaki-Raman S, Schulze A (2020). Greasing the WHEELS of the cancer machine: the role of lipid metabolism in cancer. Cell Metab.

[23] Aas V, Kase ET, Solberg R (2004). Chronic hyperglycaemia promotes lipogenesis and triacylglycerol accumulation in human skeletal muscle cells. Diabetologia.

[24] Song Z, Xiaoli AM, Yang F (2018). Regulation and metabolic significance of de novo lipogenesis in adipose tissues. Nutrients.

[25] Sena LA, Denmeade SR (2021). Fatty acid synthesis in prostate cancer: vulnerability or epiphenomenon?. Cancer Res.

[26] Butler LM, Perone Y, Dehairs J (2020). Lipids and cancer: emerging roles in pathogenesis, diagnosis and therapeutic intervention. Adv Drug Deliv Rev.

[27] Wang Q, Tan Y, Jiang T (2022). Metabolic reprogramming and its relationship to survival in hepatocellular carcinoma. Cells.

[28] Frohlich J, Mazza T, Sobolewski C (2021). GDF11 rapidly increases lipid accumulation in liver cancer cells through ALK5-dependent signaling. Biochim Biophys Acta Mol Cell Biol Lipids.

[29] Zuo X, Chen Z, Gao W (2020). M6A-mediated upregulation of LINC00958 increases lipogenesis and acts as a nanotherapeutic target in hepatocellular carcinoma. J Hematol Oncol.

[30] Wang B, Zhang H, Chen YF (2022). Acyl-CoA thioesterase 9 promotes tumour growth and metastasis through reprogramming of fatty acid metabolism in hepatocellular carcinoma. Liver Int.

[31] Batchuluun B, Pinkosky SL, Steinberg GR (2022). Lipogenesis inhibitors: therapeutic opportunities and challenges. Nat Rev Drug Discov.

[32] Che L, Chi W, Qiao Y (2020). Cholesterol biosynthesis supports the growth of hepatocarcinoma lesions depleted of fatty acid synthase in mice and humans. Gut.

[33] Wu D, Yang Y, Hou Y (2022). Increased mitochondrial fission drives the reprogramming of fatty acid metabolism in hepatocellular carcinoma cells through suppression of Sirtuin 1. Cancer Commun (Lond).

[34] Ni W, Lin S, Bian S (2020). USP7 mediates pathological hepatic de novo lipogenesis through promoting stabilization and transcription of ZNF638. Cell Death Dis.

[35] Peng JY, Cai DK, Zeng RL (2022). Upregulation of superenhancer-driven LncRNA FASRL by USF1 promotes de novo fatty acid biosynthesis to exacerbate hepatocellular carcinoma. Adv Sci (Weinh).

[36] Ning Z, Guo X, Liu X (2022). USP22 regulates lipidome accumulation by stabilizing PPARγ in hepatocellular carcinoma. Nat Commun.

[37] Han Q, Chen CA, Yang W (2021). ATP-citrate lyase regulates stemness and metastasis in hepatocellular carcinoma via the Wnt/β-catenin signaling pathway. Hepatobiliary Pancreat Dis Int.

[38] Liu HH, Xu Y, Li CJ (2022). An SCD1-dependent mechanoresponsive pathway promotes HCC invasion and metastasis through lipid metabolic reprogramming. Mol Ther.

[39] Chandrasekaran P, Weiskirchen R. The role of SCAP/SREBP as central regulators of lipid metabolism in hepatic steatosis. Int J Mol Sci. 2024;25(2):1109. 10.3390/ijms25021109 PMC10815951 38256181

[40] Ke C, Xiao C, Li J, et al. FMO2 ameliorates nonalcoholic fatty liver disease by suppressing ER-to-Golgi transport of SREBP1. Hepatology. 2023;10–1097. 10.1097/HEP.0000000000000643 37874228

[41] Rong S, Xia M, Vale G, et al. DGAT2 inhibition blocks SREBP-1 cleavage and improves hepatic steatosis by increasing phosphatidylethanolamine in the ER. Cell Metab. 2024;36(3):617–629.e7. 10.1016/j.cmet.2024.01.011 PMC10939742 38340721

[42] Su F, Koeberle A. Regulation and targeting of SREBP-1 in hepatocellular carcinoma. Cancer and Metastasis Rev. 2023;1–36. 10.1007/s10555-023-10156-5 PMC11156753 38036934

[43] Li LY, Yang Q, Jiang YY (2021). Interplay and cooperation between SREBF1 and master transcription factors regulate lipid metabolism and tumor-promoting pathways in squamous cancer. Nat Commun.

[44] Wang H, Chen Y, Liu Y (2022). The lncRNA ZFAS1 regulates lipogenesis in colorectal cancer by binding polyadenylate-binding protein 2 to stabilize SREBP1 mRNA. Mol Ther Nucleic Acids.

[45] Gabitova-Cornell L, Surumbayeva A, Peri S (2020). Cholesterol Pathway Inhibition Induces TGF-β Signaling to Promote Basal Differentiation in Pancreatic Cancer. Cancer Cell.

[46] Yamashita T, Honda M, Takatori H (2009). Activation of lipogenic pathway correlates with cell proliferation and poor prognosis in hepatocellular carcinoma. J Hepatol.

[47] Cheng C, Geng F, Li Z (2022). Ammonia stimulates SCAP/Insig dissociation and SREBP-1 activation to promote lipogenesis and tumour growth. Nat Metab.

[48] Xu D, Wang Z, Xia Y (2020). The gluconeogenic enzyme PCK1 phosphorylates INSIG1/2 for lipogenesis. Nature.

[49] Heo MJ, Kang SH, Kim YS (2020). UBC12-mediated SREBP-1 neddylation worsens metastatic tumor prognosis. Int J Cancer.

[50] Chen J, Ding C, Chen Y (2021). ACSL4 reprograms fatty acid metabolism in hepatocellular carcinoma via c-Myc/SREBP1 pathway. Cancer Lett.

[51] Yu X, Lin Q, Wu Z (2020). ZHX2 inhibits SREBP1c-mediated de novo lipogenesis in hepatocellular carcinoma via miR-24-3p. J Pathol.

[52] Liu T, Yu J, Ge C (2022). Sperm associated antigen 4 promotes SREBP1-mediated de novo lipogenesis via interaction with lamin A/C and contributes to tumor progression in hepatocellular carcinoma. Cancer Lett.

[53] Krycer JR, Sharpe LJ, Luu W (2010). The Akt-SREBP nexus: cell signaling meets lipid metabolism. Trends Endocrinol Metab.

[54] Porstmann T, Santos CR, Griffiths B (2008). SREBP activity is regulated by mTORC1 and contributes to Akt-dependent cell growth. Cell Metab.

[55] Yi J, Zhu J, Wu J (2020). Oncogenic activation of PI3K-AKT-mTOR signaling suppresses ferroptosis via SREBP-mediated lipogenesis. Proc Natl Acad Sci U S A.

[56] Chen J, Chen J, Huang J (2019). HIF-2α upregulation mediated by hypoxia promotes NAFLD-HCC progression by activating lipid synthesis via the PI3K-AKT-mTOR pathway. Aging (Albany NY).

[57] Hernandez S, Simoni-Nieves A, Gerardo-Ramírez M (2021). GDF11 restricts aberrant lipogenesis and changes in mitochondrial structure and function in human hepatocellular carcinoma cells. J Cell Physiol.

[58] Jia J, Che L, Cigliano A, et al. Pivotal role of fatty acid synthase in c-MYC driven hepatocarcinogenesis. Int J Mol Sci. 2020;21(22):8467. 10.3390/ijms21228467 PMC7696085 33187130

[59] Koundouros N, Poulogiannis G (2020). Reprogramming of fatty acid metabolism in cancer. Br J Cancer.

[60] Soukupova J, Malfettone A, Bertran E, et al. Epithelial-Mesenchymal Transition (EMT) induced by TGF-β in hepatocellular carcinoma cells reprograms lipid metabolism. Int J Mol Sci. 2021;22(11):5543. 10.3390/ijms22115543 PMC8197297 34073989

[61] Tao L, Ding X, Yan L (2022). CD36 accelerates the progression of hepatocellular carcinoma by promoting FAs absorption[J]. Med Oncol.

[62] Nath A, Li I, Roberts LR (2015). Elevated free fatty acid uptake via CD36 promotes epithelial-mesenchymal transition in hepatocellular carcinoma. Sci Rep.

[63] Tian Y, Yang B, Qiu W (2019). ER-residential Nogo-B accelerates NAFLD-associated HCC mediated by metabolic reprogramming of oxLDL lipophagy. Nat Commun.

[64] Hong J, Liu J, Zhang Y (2023). MiR-3180 inhibits hepatocellular carcinoma growth and metastasis by targeting lipid synthesis and uptake. Cancer Cell Int.

[65] Wang H, Liu F, Wu X (2024). Cancer-associated fibroblasts contributed to hepatocellular carcinoma recurrence and metastasis via CD36-mediated fatty-acid metabolic reprogramming. Exp Cell Res.

[66] Li Z, Liao X, Hu Y (2023). SLC27A4-mediated selective uptake of mono-unsaturated fatty acids promotes ferroptosis defense in hepatocellular carcinoma. Free Radic Biol Med.

[67] Bogie JFJ, Haidar M, Kooij G (2020). Fatty acid metabolism in the progression and resolution of CNS disorders. Adv Drug Deliv Rev.

[68] Ruiz De Gauna M, Biancaniello F, González-Romero F (2022). Cholangiocarcinoma progression depends on the uptake and metabolization of extracellular lipids. Hepatol.

[69] Wang T, Fahrmann JF, Lee H (2018). JAK/STAT3-regulated fatty acid β-oxidation is critical for breast cancer stem cell self-renewal and chemoresistance. Cell Metab.

[70] Longo N, Di San A, Filippo C, Pasquali M (2006). Disorders of carnitine transport and the carnitine cycle. Am J Med Genet C Semin Med Genet.

[71] Jogl G, Hsiao YS, Tong L (2004). Structure and function of carnitine acyltransferases. Ann N Y Acad Sci.

[72] Liu Z, Liu W, Wang W (2023). CPT1A-mediated fatty acid oxidation confers cancer cell resistance to immune-mediated cytolytic killing. Proc Natl Acad Sci USA.

[73] Lin J, Zhang P, Liu W (2023). A positive feedback loop between ZEB2 and ACSL4 regulates lipid metabolism to promote breast cancer metastasis. Elife.

[74] Zhang T, Zhang Y, Liu J (2022). MicroRNA-377-3p inhibits hepatocellular carcinoma growth and metastasis through negative regulation of CPT1C-mediated fatty acid oxidation. Cancer Metab.

[75] Ren M, Xu H, Xia H (2021). Simultaneously targeting SOAT1 and CPT1A ameliorates hepatocellular carcinoma by disrupting lipid homeostasis. Cell Death Discov.

[76] González-Romero F, Mestre D, Aurrekoetxea I (2021). E2F1 and E2F2-mediated repression of CPT2 establishes a lipid-rich tumor-promoting environment. Cancer Res.

[77] Gao Q, Zhang G, Zheng Y (2020). SLC27A5 deficiency activates NRF2/TXNRD1 pathway by increased lipid peroxidation in HCC. Cell Death Differ.

[78] Yuan P, Mu J, Wang Z (2021). Down-regulation of SLC25A20 promotes hepatocellular carcinoma growth and metastasis through suppression of fatty-acid oxidation. Cell Death Dis.

[79] Ma Y, Temkin SM, Hawkridge AM (2018). Fatty acid oxidation: An emerging facet of metabolic transformation in cancer. Cancer Lett.

[80] Ma APY, Yeung CLS, Tey SK (2021). Suppression of ACADM-mediated fatty acid oxidation promotes hepatocellular carcinoma via aberrant CAV1/SREBP1 signaling. Cancer Res.

[81] Guo D, Zhang X, Cui H (2022). ACADL functions as a tumor suppressor in hepatocellular carcinoma metastasis by inhibiting matrix metalloproteinase 14. Front Oncol.

[82] Kubes P, Jenne C (2018). Immune responses in the liver. Annu Rev Immunol.

[83] Xia L, Oyang L, Lin J (2021). The cancer metabolic reprogramming and immune response. Mol Cancer.

[84] Masetti M, Carriero R, Portale F (2022). Lipid-loaded tumor-associated macrophages sustain tumor growth and invasiveness in prostate cancer. J Exp Med.

[85] Storz P (2023). Roles of differently polarized macrophages in the initiation and progressionof pancreatic cancer. Front Immunol.

[86] Mantovani A, Sozzani S, Locati M (2002). Macrophage polarization: tumor-associated macrophages as a paradigm for polarized M2 mononuclear phagocytes. Trends Immunol.

[87] Dussold C, Zilinger K, Turunen J (2024). Modulation of macrophage metabolism as an emerging immunotherapy strategy for cancer. J Clin Invest.

[88] Jääskeläinen MM, Tumelius R, Hämäläinen K (2024). High numbers of CD163+ tumor-associated macrophages predict poor prognosis in HER2+ breast cancer. Cancers (Basel).

[89] Su P, Wang Q, Bi E (2020). Enhanced lipid accumulation and metabolism are required for the differentiation and activation of tumor-associated macrophages. Cancer Res.

[90] Wu L, Zhang X, Zheng L (2020). RIPK3 orchestrates fatty acid metabolism in tumor-associated macrophages and hepatocarcinogenesis. Cancer Immunol Res.

[91] Liu S, Zhang H, Li Y (2021). S100A4 enhances protumor macrophage polarization by control of PPAR-γ-dependent induction of fatty acid oxidation. J Immunother Cancer.

[92] Zhang J, Zhang H, Ding X (2022). Crosstalk between macrophage-derived PGE(2) and tumor UHRF1 drives hepatocellular carcinoma progression. Theranostics.

[93] Liu L, Liu Z, Gao J (2022). CD8+ T cell trajectory subtypes decode tumor heterogeneity and provide treatment recommendations for hepatocellular carcinoma. Front Immunol.

[94] Schmidt NM, Wing PAC, Diniz MO (2021). Targeting human Acyl-CoA:cholesterol acyltransferase as a dual viral and T cell metabolic checkpoint. Nat Commun.

[95] Tiwary S, Berzofsky JA, Terabe M (2019). Altered lipid tumor environment and its potential effects on nkt cell function in tumor immunity. Front Immunol.

[96] Tang W, Zhou J, Yang W (2022). Aberrant cholesterol metabolic signaling impairs antitumor immunosurveillance through natural killer T cell dysfunction in obese liver[J]. Cell Mol Immunol.

[97] Cheng X, Tan X, Wang W (2023). Long-Chain Acylcarnitines induce senescence of invariant natural killer t cells in hepatocellular carcinoma. Cancer Res.

[98] Xie M, Lin Z, Ji X (2023). FGF19/FGFR4-mediated elevation of ETV4 facilitates hepatocellular carcinoma metastasis by upregulating PD-L1 and CCL2. J Hepatol.

[99] Wang N, Tan HY, Lu Y (2021). PIWIL1 governs the crosstalk of cancer cell metabolism and immunosuppressive microenvironment in hepatocellular carcinoma. Signal Transduct Target Ther.

[100] Santos PM, Menk AV, Shi J (2019). Tumor-derived α-fetoprotein suppresses fatty acid metabolism and oxidative phosphorylation in dendritic cells. Cancer Immunol Res.

[101] Esquejo RM, Roqueta-Rivera M, Shao W (2021). Dipyridamole inhibits lipogenic gene expression by retaining SCAP-SREBP in the endoplasmic reticulum. Cell Chem Biol.

[102] Meng H, Shen M, Li J (2021). Novel SREBP1 inhibitor cinobufotalin suppresses proliferation of hepatocellular carcinoma by targeting lipogenesis. Eur J Pharmacol.

[103] Huang CJ, Zhang CY, Zhao YK (2023). Bufalin inhibits tumorigenesis and SREBP-1-mediated lipogenesis in hepatocellular carcinoma via modulating the ATP1A1/CA2 axis. Am J Chin Med.

[104] Cheng L, Deepak R, Wang G (2023). Hepatic mitochondrial NAD + transporter SLC25A47 activates AMPKα mediating lipid metabolism and tumorigenesis. Hepatology.

[105] Zhao X, Qin W, Jiang Y (2020). ACADL plays a tumor-suppressor role by targeting Hippo/YAP signaling in hepatocellular carcinoma. NPJ Precis Oncol.

[106] Zhou Y, Guo Y, Zhu Y (2021). Dual PPARγ/ɑ agonist oroxyloside suppresses cell cycle progression by glycolipid metabolism switch-mediated increase of reactive oxygen species levels. Free Radic Biol Med.

[107] Wang H, Zhou Y, Xu H (2022). Therapeutic efficacy of FASN inhibition in preclinical models of HCC. Hepatology.

[108] Man S, Yao J, Lv P (2020). Curcumin-enhanced antitumor effects of sorafenib via regulating the metabolism and tumor microenvironment. Food Funct.

[109] Mo Y, Wu Y, Li X (2020). Osthole delays hepatocarcinogenesis in mice by suppressing AKT/FASN axis and ERK phosphorylation. Eur J Pharmacol.

[110] Mullen GE, Yet L (2015). Progress in the development of fatty acid synthase inhibitors as anticancer targets. Bioorg Med Chem Lett.

[111] Mabrouk N, Lecoeur B, Bettaieb A, et al. Impact of Lipid Metabolism on Antitumor Immune Response. Cancers (Basel), 2022, 14(7). 10.3390/cancers14071850 PMC8997602 35406621

